# 4106 Personalizing Care For Colorectal Cancer: Identifying Novel Opportunities

**DOI:** 10.1017/cts.2020.340

**Published:** 2020-07-29

**Authors:** Zachary Rivers, David Stenehjem, Emil Lou, Andrew Nelson, Pamala Jacobson, Karen Kuntz

**Affiliations:** 1 University of Minnesota CTSI; 2 University of Minnesota

## Abstract

OBJECTIVES/GOALS: This project seeks to understand how personalized medicine can optimize care for patients with colorectal cancer. It identifies opportunities for personalized medicine to improve clinical outcomes, and uses cost-effectiveness analysis to assess the clinical and financial impact of this approach. METHODS/STUDY POPULATION: This project uses two methods to understand the impact of personalized medicine. First, this project has used SEER-Medicare data in conjunction with Clinical Pharmacogenetics Implementation Consortium guidelines to identify medications used by patients with colorectal cancer that can be impacted by genetic variants. This data will then be combined with population genetic variant rates to understand the likely impact screening for a given variant will have on medication response and adverse events. Medication use frequencies and genetic variant rates are then used to populate cost-effectiveness models that simulate the clinical and financial outcomes, identifying optimal genes to screen. RESULTS/ANTICIPATED RESULTS: The first result will be a comprehensive overview of treatment patterns for patients with colorectal cancer in the United States, as well as the treatments used for disease-induced comorbidities. The second result will be the identification of genetic variants based on population rates and medication utilization that should be screened in this patient population. The final result will be a breakdown of the clinical and financial outcomes associated with implementing screening for the identified genes. Preliminary results from a two-gene cost-effectiveness analysis demonstrates that screening for variants in those genes improves both clinical and financial outcomes. DISCUSSION/SIGNIFICANCE OF IMPACT: This project demonstrates how current treatment approaches can be optimized via personalized medicine. It uses epidemiological methods to identify opportunities to integrate genetic findings from other diseases, and uses cost-effectiveness analysis to understand the impact of transforming care. CONFLICT OF INTEREST DESCRIPTION: Stocks-Aurinia, Syndax, Adaptimmune, Rigel pharma

